# Fluorescence Characteristics of Aqueous Synthesized Tin Oxide Quantum Dots for the Detection of Heavy Metal Ions in Contaminated Water

**DOI:** 10.3390/nano9091294

**Published:** 2019-09-10

**Authors:** Jianqiao Liu, Qianru Zhang, Weiting Xue, Haipeng Zhang, Yu Bai, Liting Wu, Zhaoxia Zhai, Guohua Jin

**Affiliations:** 1 College of Information Science and Technology, Dalian Maritime University, Linghai Road 1, Ganjingzi District, Dalian 116026, China; 2 Institute of Agriculture Resources and Regional Planning, Chinese Academy of Agricultural Sciences, Beijing 100081, China

**Keywords:** tin oxide, quantum dot, heavy metal ion, water pollution, fluorescence, sensing mechanism

## Abstract

Tin oxide quantum dots were synthesized in aqueous solution via a simple hydrolysis and oxidation process. The morphology observation showed that the quantum dots had an average grain size of 2.23 nm. The rutile phase SnO_2_ was confirmed by the structural and compositional characterization. The fluorescence spectroscopy of quantum dots was used to detect the heavy metal ions of Cd^2+^, Fe^3+^, Ni^2+^ and Pb^2+^, which caused the quenching effect of photoluminescence. The quantum dots showed the response of 2.48 to 100 ppm Ni^2+^. The prepared SnO_2_ quantum dots exhibited prospective in the detection of heavy metal ions in contaminated water, including deionized water, deionized water with Fe^3+^, reclaimed water and sea water. The limit of detection was as low as 0.01 ppm for Ni^2+^ detection. The first principle calculation based on the density function theory demonstrated the dependence of fluorescence response on the adsorption energy of heavy metal ions as well as ion radius. The mechanism of fluorescence response was discussed based on the interaction between Sn vacancies and Ni^2+^ ions. A linear correlation of fluorescence emission intensity against Ni^2+^ concentration was obtained in the logarithmic coordinates. The density of active Sn vacancies was the crucial factor that determined fluorescence response of SnO_2_ QDs to heavy metal ions.

## 1. Introduction

Water pollution has become a grave concern throughout the world. Industrial wastewater discharge heavy metals, which are produced from factories fabricating metals, papers and chemicals [1,2,3]. These heavy metal ions bring severe risks to living creatures because most of them are non-biodegradable and toxic even at trace levels [4,5,6]. Most heavy metals are non-biodegradable and may accumulate in the aquatic and plant organisms [7]. The danger caused by heavy metals are biomagnified in the food web [8]. Fe^3+^ ions are one of the most fundamental elements in body and the excess of Fe^3+^ from the permissible limit could result in several severe diseases [9]. Pb^2+^ of extremely low concentration increases the risks of cardiovascular disease and cancer, especially in children [10]. It is toxic to cells after the interaction with calcium and zinc proteins, which are important in the process of cell signal transmission and gene expression [11,12,13]. Cd^2+^ has a high transferability from environment to plants by the absorption through roots, causing withered or dead plants [14,15]. High concentration intake of Ni^2+^ causes skin dermatitis, nausea, chronic asthma, coughing and cancer [16].

Therefore, it is urgent to develop a technique that is of high response, low limit of detection, simplicity in operation and capability of in situ detection. There are a few of techniques to detect heavy metal ions, such as atomic absorption spectroscopy (AAS) [17], inductively coupled plasma mass spectrometry (ICP-MS) [18] and fluorescence spectroscopy (FS) [19,20]. The quantum dot (QD) method, with grain size less than 5 nm, has fluorescence effects, which could be used in the detection of drugs [21], anions [22] and organic pollutants [23]. It also has prospects in the detection of heavy metal ions by using its characteristics of fluorescence.

The effect of fluorescence is a process where a QD emits light after it has absorbed electromagnetic radiation from a excitation source. The electrons in valence band are stimulated to reach conduction band by the excitation light. Then, when the electrons transit from conduction band to valence band, the fluorescence emission takes place as a kind of energy release [24]. It has been found that the fluorescence emission could be influenced by many factors, and one of them is the presence of heavy metal ions, which caus the quenching of fluorescence [25]. The ions of heavy metals can adsorb the released energy of the electron transition, interfering with the fluorescence emission. Therefore, the fluorescence nature of semiconductor QDs has been put into practice of heavy metal ion detection, even though the mechanism of fluorescence quenching is still of high complexity.

Several kinds of QDs have been synthesized, such as CdS [23], CdTe [26], ZnS [27] and their composites [28,29]. However, some of them contain toxic elements in themselves. The tin oxide (SnO_2_) QD is an environment-friendly semiconductor. It has the advantages of non-toxicity, chemical stability and low cost. Based on the optical characteristics, SnO_2_ photoluminescence sensors were developed for xanthene dyes [30], DNA [31] and 1,4 Bis ((2-Methyl) thio) Phenylamino methyl benzene Schiff [32]. However, there have been few reports of heavy metal ion detection by SnO_2_ sensors. There are some techniques to prepare SnO_2_ QDs. However, the organic reagents of oleic acid, toluene, oleylamine and hydrazine need to be used in the fabrication [33,34,35]. The organic compounds are harmful to human beings and the environment. They also increase the risk of operator poisoning and the cost of environmental remediation in factories. Therefore, it is expected to find a route to prepare SnO_2_ QDs with simple, cheap and environment-friendly fabrication. Furthermore, these SnO_2_ QDs are potentially applicable to the detection of heavy metal ions in contaminated water.

In the present work, SnO_2_ QDs were synthesized in the aqueous solution for the detection of heavy metal ions. Several characterizations were used to confirm the structure and composition of the prepared QDs. The detection of heavy metal ions was completed by using the fluorescence effect of QDs, which is quenched by the ions of Cd^2+^, Fe^3+^, Ni^2+^ and Pb^2+^. The mechanism of fluorescence quenching by the heavy metal ions is discussed in combination with the first principle calculation based on the density function theory.

## 2. Materials and Methods

SnO_2_ QDs were synthesized in the aqueous solution by a facial method [36]. Analytical reagents of SnCl_2_·2H_2_O (≥98.0%, Sinopharm Chemical Reagent Co., Ltd., Shanghai, China) and thiourea (CH_4_N_2_S, ≥99.0%, Sinopharm Chemical Reagent Co., Ltd., Shanghai, China) were used as raw materials. 2.257 g SnCl_2_·2H_2_O and 0.077 g CH_4_N_2_S were dissolved into deionized water of 50 mL. The stannous chloride transformed to stannous hydroxide during hydrolysis process and the suspension was stirred in a magnet stirring apparatus for 24 h at room temperature. In this process, the thiourea, as an accelerator, promoted the forward reaction by consuming HCl in the solution, as shown in Equation (1). Meanwhile, stannous hydroxide was oxidized by aerial oxygen with SnO_2_ QDs obtained, as shown in Equation (2):

(1) $${\mathrm{SnCl}}_{2}+2H_{2}O↔{\mathrm{Sn}(\mathrm{OH})}_{2}+2\mathrm{HCl}$$

(2) $${2\mathrm{Sn}(\mathrm{OH})}_{2}+O_{2}\to {2\mathrm{SnO}}_{2}+2H_{2}O$$

Thus, the aqueous SnO_2_ QD solution was acquired after the completion of hydrolysis and oxidation. The grain size and Zeta potential of the SnO_2_ QDs were analyzed by dynamic light scattering (DLS, Malvern Zetasizer Nano ZS 90, Malvern panalytical Ltd., Malvern, UK). The morphology was observed by high resolution transmission electron microscopy (HRTEM, JEM-3200FS, JEOL, Tokyo, Japan). The solution was dried to powder for X-ray diffraction (XRD, Rigaku D/MAX-Ultima, Rigaku, Tokyo, Japan) and X-ray photoelectron spectroscopy (XPS, Thermo Scientific ESCALAB 250 XI, ThermoFisher Scientific, Waltham, MA, USA). A fluorescence spectrometer (FLS-980, Edinburgh Instruments, Edinburgh, UK) was used to characterize the fluorescence performances of the SnO_2_ QDs. The wavelength of excitation in fluorescence characterization was 280 nm. The SnO_2_ QD solution was diluted to 0.002 mol/L of Sn atoms and it was incorporated with heavy metal ions of Cd^2+^, Fe^3+^, Ni^2+^ as well as Pb^2+^. The fluorescence response (*S*) was defined as the ratio of the maximum intensity of SnO_2_ QDs (*F*_0_) to the one of SnO_2_ QDs with heavy metal ion incorporation (*F*), as *S* = *F*_0_/*F*. The characterization of fluorescence was carried out immediately after the incorporation. In order to evaluate the applicability of SnO_2_ QDs in the practical detection of heavy metal ions, several types of background water were employed, namely, deionized water, reclaimed water and sea water. The sea water was collected from Xinghai Bay of Yellow Sea, Dalian, China. There were no Ni^2+^ ions in the deionized water and reclaimed water. The reclaimed water contained cations of Fe^3+^ (<0.3 mg/L) and Mn^2+^ (<0.1 mg/L) as well as several anions (<1.0 mg/L). The main composition of sea water included cations of Na^+^ (11.04 mg/L), K^+^ (0.40 mg/L), Ca^2+^ (0.42 mg/L) and Mg^2+^ (1.33 mg/L) as well as anions of Cl^−^ (19.86 mg/L) and SO_4_^2−^ (2.77 mg/L). Ni^2+^ was not taken into account because it was insignificant compared to other major solutes.

## 3. Results and Discussion

### 3.1. Structure and Morphology

**Figure 1:**
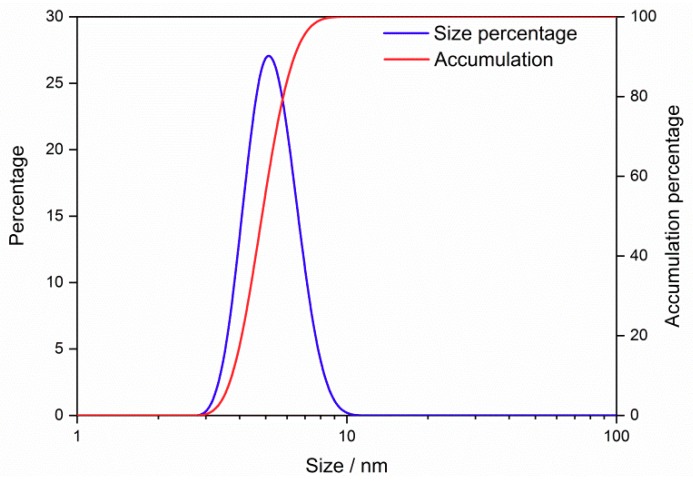
The grain size distribution of the SnO_2_ quantum dots in aqueous solution.

**Figure 2:**
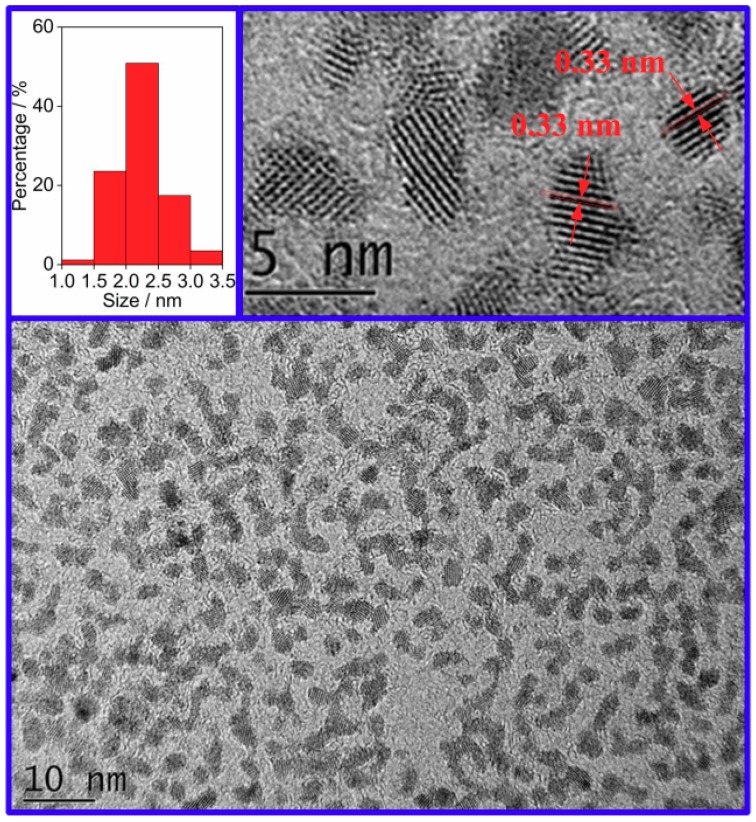
High resolution transmission electron microscopy image of the SnO_2_ quantum dots in the aqueous solution.

The grain size distribution of the SnO_2_ QDs from DLS is shown in Figure 1. The grain size was from 2 to 10 nm. The peak appeared at 5.3 nm and approximate 90% of the grains were of the size within 4–6 nm. The morphology of the SnO_2_ QDs was observed by HRTEM, as shown in Figure 2. The prepared QDs have a uniform dispersion in the aqueous solution. The Zeta potential of the as-prepared sample was 17.3 mV and it was measured to be 17.1 mV after 3 months of storage at room temperature. Thus, the SnO_2_ QDs were stable in the aqueous solution for several months. The average grain size was measured to be 2.23 nm. The grain sizes of QDs were from 1.4 nm to 3.4 nm, and over 50% of them were between 2.0 to 2.5 nm. The characteristic spacing of 0.33 nm was observed, corresponding to the (110) planes of the rutile phase of SnO_2_.

**Figure 3:**
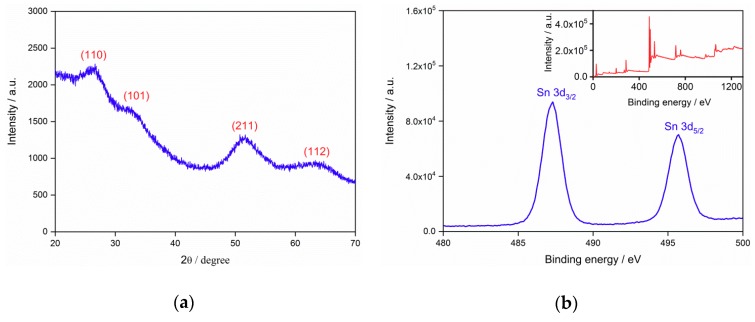
(**a**) X-ray diffraction (XRD) pattern of the SnO_2_ quantum dot powder; (**b**) X-ray photoelectron spectroscopy (XPS) spectrum of the SnO_2_.

Figure 3a shows the XRD pattern of the SnO_2_ powder, which was obtained from the dried aqueous solution with SnO_2_ QDs. Four main peaks of (110), (101), (211) and (112) were observed, in agreement with the rutile phase of SnO_2_. The lattice constants of SnO_2_ unit cells were evaluated to be *a* = *b* = 4.74677 Å and *c* = 3.18196 Å. The crystallite size of the QDs was calculated to be 2.3 nm according to the Scherrer’s formula. The size coincided with the grain size observed from HRTEM, but was smaller than the result of DLS size distribution in Figure 1. The deviation may be ascribed to the aggregation of QDs, which affected the light scattering during the DLS measurement. The XPS spectrum of the SnO_2_ QDs is shown in Figure 3b, which shows the presence of C 1s, O 1s and Sn 3d. The high solution pattern of Sn 3d shows two peaks of 487.3 eV and 495.6 eV, corresponding to the Sn 3d_3/2_ and Sn3d_5/2_, respectively. The spectrum is in good agreement with the standard pattern from the rutile SnO_2_ sample [37,38].

### 3.2. Fluorescence Response to Heavy Metal Ions

**Figure 4:**
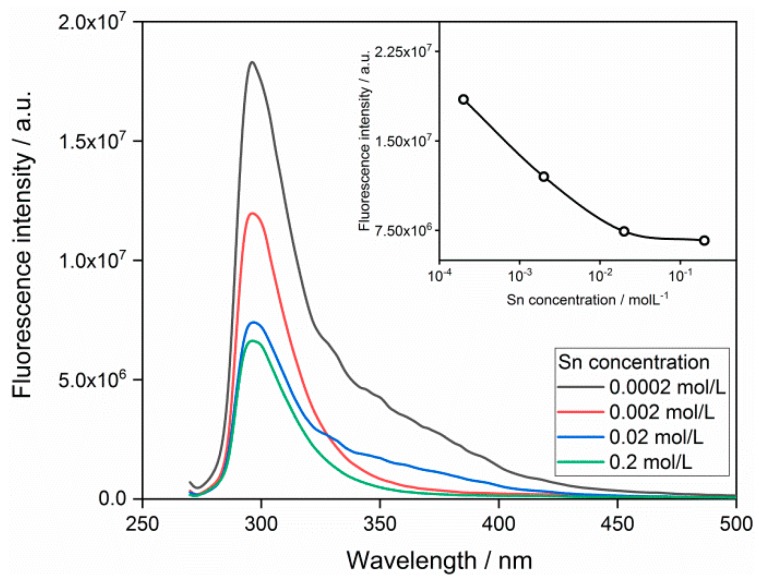
Dependence of fluorescence emission on the concentration of SnO_2_ quantum dots.

**Figure 5:**
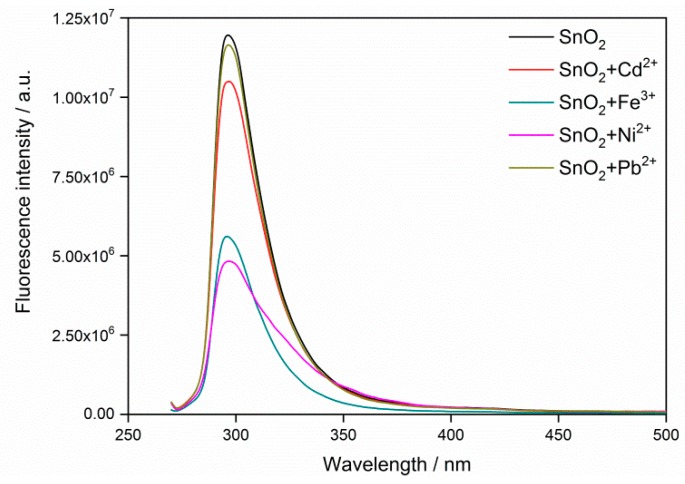
Fluorescence spectrum of SnO_2_ quantum dots before and after the incorporation of heavy metal ions.

**Figure 6:**
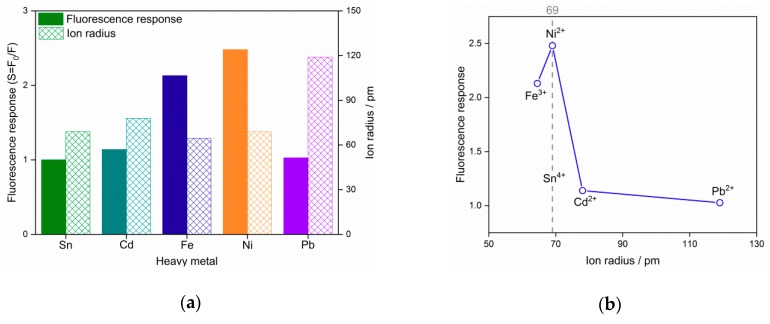
(**a**) Fluorescence response of the SnO_2_ quantum dots and ion radius of various types of heavy metals; (**b**) dependence of fluorescence response on the ion radius of heavy metals.

The fluorescence spectra of SnO_2_ QDs with various concentrations are shown in Figure 4, where the peak appears at the emission wavelength of 300 nm. Considering the excitation wavelength of 280 nm, the present SnO_2_ QDs show a low Stocks shift. The self-quenching property of QDs was observed when the Sn concentration increased. The heavy metal ion solutions of 100 ppm were incorporated with the SnO_2_ QDs and the incorporations result in the quenching of fluorescence, as shown in Figure 5. It was observed that Ni^2+^ and Fe^3+^ perform stronger attenuations to the fluorescence emission than other heavy metals. Figure 6a illustrates the fluorescence responses of SnO_2_ QDs to the various heavy metal ions. The QDs show responses to all the heavy metals, which may interact with QDs by interfering the electronic transition from conduction band to valence band. The Ni^2+^ incorporation stimulates the highest response of 2.48.

The detection of heavy metal ions is completed by the quenching of fluorescence. The quenching mechanism has been proven to be complex [39,40], in which the heavy metal ions interfere with the fluorescence emission by adsorbing the energy of electron transition from conduction band to valence band. The prepared SnO_2_ QDs have an emission peak at 300 nm, in agreement with the previous report [41]. The position of the emission peak remained the same after the incorporation of heavy metals, revealing that the band gap had not shifted. Thus, the fluorescence quenching may result from the variation of surface states of the QDs. The radius of Sn^4+^ ion was found to be 69 pm, while the heavy metals of Cd^2+^, Fe^3+^, Ni^2+^ and Pb^2+^ have the ion radii of 78, 64.5, 69 and 119 pm [42], as shown in Figure 6a,b. It was found that the radius difference between heavy metal ions with Sn^4+^ had the same relationship with the response of fluorescence quenching. The Ni^2+^ ion had the same radius as the Sn^4+^ ion and this could promote its interaction with the QD surface, where the crystal lattice is lacking in integrity. Therefore, Ni^2+^ ions perform as surface states on QDs and cause a strong fluorescence quenching. For the other heavy metal ions, their radii differed from Sn^4+^ and the radius difference would bring mismatches in crystal lattice when they interact with QD surface. Hence, the density of surface states would be limited, leaving the little fluorescence responses.

**Figure 7:**
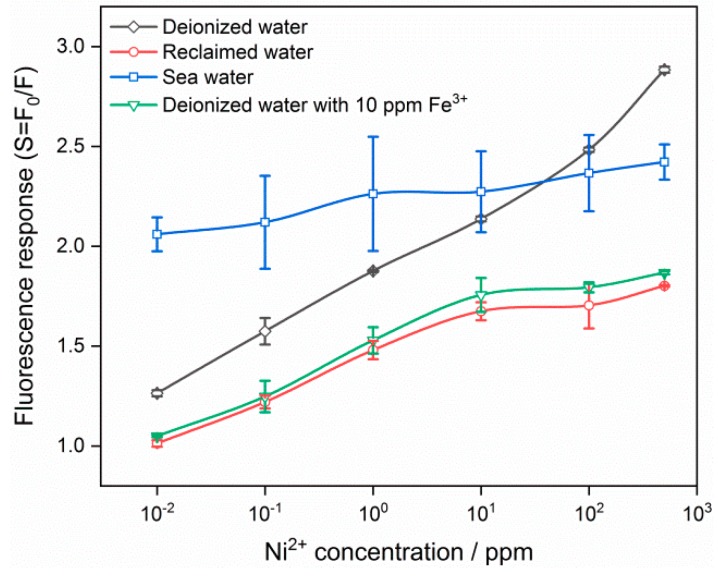
Fluorescence response of SnO_2_ quantum dots to Ni^2+^ ions of 0.01 to 500 ppm in the background solutions of deionized water, deionized water with 10 ppm Fe^3+^, reclaimed water and sea water.

Figure 7 shows the fluorescence response of SnO_2_ QDs to Ni^2+^ with concentration of 0.01–500 ppm in the background solutions of deionized water, deionized water with 10 ppm Fe^3+^, reclaimed water and sea water. In the deionized water, the QDs reveal positive dependence of fluorescence response with Ni^2+^ concentration. The concentration linear range is from 10^−2^ to 500 ppm in the logarithmic coordinates. If the sensitivity is defined as the slope of fluorescence response against target pollutant concentration in the logarithmic coordinates, it is evaluated to be 0.073 for SnO_2_ QDs in deionized water for Ni^2+^ detection. However, the response appears to be degraded in the presence of 10 ppm Fe^3+^, which is competitive to Ni^2+^ in the deionized water. A similar dependence was also observed for the fluorescence response of QDs in reclaimed water, because it contains impurities of Fe^3+^ and Mn^2+^. They were also responsible for the non-linear correlation at the high Ni^2+^ concentration over 100 ppm. The limit of detection is the least concentration of target pollutant, which can stimulate detectable fluorescence response of SnO_2_ QDs. As shown in Figure 7, the limits of detection are 0.01 ppm with the responses of 1.26 and 1.01 in deionized water and reclaimed water, respectively. The fluorescence illustrated a different performance in case of the sea water background. The response is between 2.06 and 2.42 within the Ni^2+^ concentration range of 0.01–500 ppm, being less sensitive to the target ion. It would be ascribed to the cations of Na^+^, K+, Ca^2+^ and Mg^2+^ as well as anions of Cl^−^ and SO_4_^2^^−^, which may interfere with the detection of Ni^2+^ by their competitive interaction with SnO_2_ QDs.

Apart from the present SnO_2_ QDs, the Ni^2+^ detection has to be completed by other QDs in a variety of circumstances. Hydrophobic core/shell CdSe/ZnS QDs were used for Ni^2+^ sensing in organic solvent. A similar non-linear dependence of fluorescence response on Ni^2+^ concentration was observed [43]. In CdS QD sensors, the correlation was found to be linear at low concentration and it became non-linear when a high Ni^2+^ concentration was introduced [44]. Furthermore, the sensing performance was found to be dependent on pH condition [45]. Thioglycolic acid capped CdTe semiconductor in aqueous solution was applied as the fluorescence sensor, which could determine Ni^2+^ without any tangible influence of a few interfering ions [46]. Imidazole modified carbon dots were also employed for photoluminescence sensors with limit of detection of 0.93 × 10^−3^ mol/L [47]. Compared to those sensors, the present SnO_2_ QDs show promising properties to develop photoluminescence sensors, which take advantages of the chemical stability, non-toxicity and low cost. On the other hand, highly sensitive spectroscopic techniques, such as inductively coupled plasma mass spectrometry (ICP-MS), inductively coupled plasma optic emission spectrometry (ICP-OES) and square wave anodic stripping voltammetric (SWASV), provide precise results with low limit of detection [48,49,50]. For example, the limits of detection of ICP-OES are 1.2, 1.1, 1.0 and 6.3 µg/L for Cu^2+^, Zn^2+^, Cd^2+^ and Ni^2+^, respectively [48]. However, these techniques are usually of high cost and need complex operations by experienced staffs. The present photoluminescence sensor of SnO_2_ QDs would be beneficial to the design of in situ devices for heavy metal ions in contaminated water.

It is noted that selectivity is an essential characteristics in the detection of heavy metal ions, because they usually coexist in the contaminated water. However, the present SnO_2_ QDs show a fluorescence response to all of them. It is necessary to develop the ability to discriminate among heavy metal ions for a practical sensor. The technique using a neural network is one of the candidates for the potential device, which contains a sensor array with various sensing properties, e.g., sensors with various Sn concentrations. Each sensor in the array would be trained by a series of fluorescence responses to an individual type of heavy metal ion or a mixture of them. Then, the device would acquire the ability of discrimination to a specific type of heavy metal ion after a group of fluorescence responses is collected. In addition, the heavy metal ions of Cd^2+^, Fe^3+^, Ni^2+^ and Pb^2+^ were investigated in the present work because they are typical pollutants in the environment. These heavy metal ions are representative pollutants, which were used to develop SnO_2_ QD photoluminescence sensors. Other heavy metal ions, such as Cu^2+^, Zn^2+^, Fe^2+^, Mn^2+^ and Hg^2+^, will need to be investigated as target pollutants in further researches. There were four different background solutions in the present work, including deionized water, deionized water with 10 ppm Fe^3+^, reclaimed water and sea water. All of them were used to check the validity of SnO_2_ QDs in the detection of Ni^2+^. Although the prepared QDs were able to detect Ni^2+^ ions as a photoluminescence sensor, the sensor performances are different in those background solutions. Therefore, it was necessary to study the SnO_2_ QD properties in a diversity of background solutions so that it can be put into practical use.

It is known that the Stern-Volmer relationship describes the dependence of fluorescence response to the concentration of quencher, as *S* = *F*_0_/*F*= 1+*k*_q_*τ*_0_ [Q]. Here, *k*_q_ is the quencher rate coefficient and *τ*_0_ is the lifetime of the emissive excited state without a quencher present. [Q] is the concentration of the quencher Q. Therefore, *S* is of linear dependence with [Q] provided that *k*_q_ and *τ*_0_ are constants. The Stern-Volmer relationship is established based on a model involving double molecules. However, the intermolecular deactivation differs from the present detection, where several individual heavy metal ions interact with a QD assembled by a matrix of super cells. Therefore, the results in Figure 7 deviate from the linear correlation and the sensing mechanism needs further discussions.

### 3.3. First Principle Calculation

**Figure 8:**
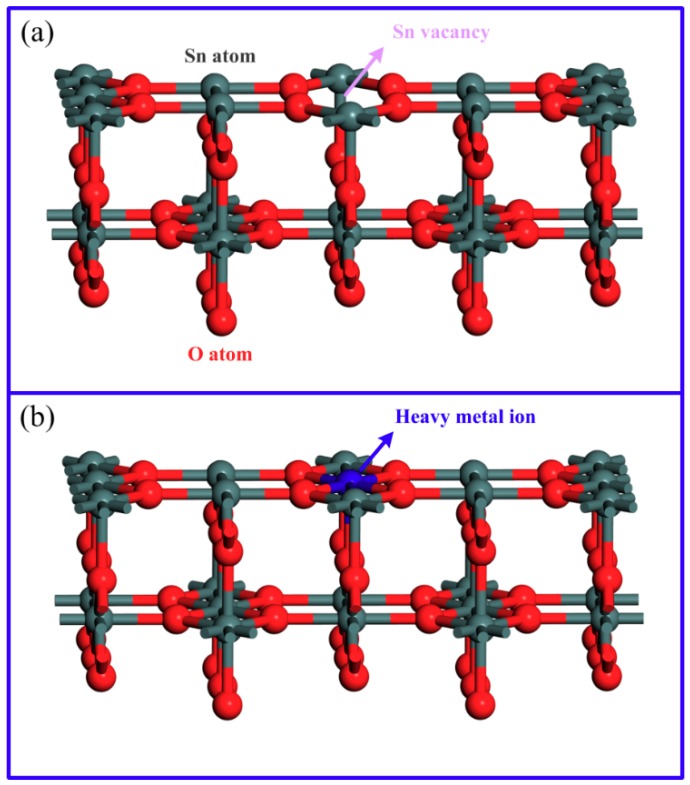
Structural model of rutile SnO_2_ system with one Sn vacancy (**a**) before and (**b**) after interaction with heavy metal ions. Atoms of each element are indicated by colors: gray for Sn, red for O and blue for heavy metal ions.

A first principle calculation has been employed for further discussion of the interaction between SnO_2_ QDs and heavy metal ions. The calculation was implemented based on the density function theory (DFT) in the Cambridge sequential total energy package (CASTEP) [51]. The Perdew-Burke-Ernzerhof (PBE) function was used to describe the exchange-correlation interaction in the generalized-gradient approximation (GGA). The structural model was established based on rutile SnO_2_ tetragonal unit cells with lattice constants of *a* = *b* = 4.7373 Å and *c* = 3.1864 Å [52]. The (110) plane with the lowest energy [53,54,55] was chosen as the surface interacted with heavy metal ions. The crystal plane was cleaved from the optimized system of SnO_2_, which contained 2 × 2 × 2 matrix of unit cells. A vacuum region of 10 Å was also employed to prevent the interaction between adjacent layers. The Brillouin zone was sampled using a 2 × 2 × 1 k-point Monkhorst-Pack mesh. In order to calculate the adsorption energy of heavy metal ions on SnO_2_ surface, one Sn atom was removed from the (110) crystal surface for a point defect as the adsorption site, as shown in Figure 8a. The system energy was indicated by *E_def_*. Then, the heavy metal ions were introduced and one of them was adsorbed on the site of defect, as shown in Figure 8b. The system energy after interaction was denoted by *E_metal_*. Thus, the adsorption energy (*E_ads_*) of the specific heavy metal ion on the SnO_2_ QD surface could be calculated according to Equation (3):

(3) $$E_{ads}=E_{metal}-E_{def}$$

The calculation results of adsorption energy are listed in Table 1. Fe^3+^ and Ni^2+^ show large *E_ads_* values of 13.46 and 7.65 eV, while the adsorption energies for Cd^2+^ and Pb^2+^ are quite low. Therefore, the Fe^3+^ and Ni^2+^ ions are much easier to be interacted with SnO_2_ QDs and the conclusion is in agreement with the correlation between fluorescence response and ion radius. It was concluded that the fluorescence performance is dependent on the adsorption energy of heavy metal ions. However, an inverse correlation was observed between adsorption energy and fluorescence response for Fe^3+^ and Ni^2+^. It is known that the fluorescence response is the consequence of the quenching of energy emission during the transition of electrons from the conduction band to the valence band. There are several ways to release the energy, such as self-quenching, resonance energy transfer (RET), exciton coupling and photoinduced electron transfer (PET) [25]. Among these, PET involves the transfer of electrons between an excited fluorescence agent and a ground state species creating a charge separation [56]. When adsorbed on the site of Sn vacancy, divalent Ni^2+^ ions may have a stronger interference to PET than trivalent Fe^3+^ ions. Therefore, the SnO_2_ QDs show a more significant fluorescence to Ni^2+^ even though Fe^3+^ has greater adsorption energy.

### 3.4. Mechanism of Fluorescence Response

As shown in the structural model of the SnO_2_ system in Figure 8, the interaction between Ni^2+^ and SnO_2_ QDs is described by a Ni^2+^ ion entering a Sn vacancy on the QD surface. It is known that a Sn vacancy acts as an acceptor in SnO_2_ system and its ionization can be expressed by the Kroger-Vink notation, as shown in Equation (4):

(4) $$V_{Sn}+4e^{\prime }↔V_{Sn}{}^{⁗}$$

where *V_Sn_* and *V_Sn_*⁗ are the Sn vacancy before and after ionization and e′ represents a free electron in the SnO_2_ grain. After being adsorbed on the SnO_2_ QD, the Ni^2+^ interacts with the Sn vacancy, as expressed in Equation (5):

(5) $$V_{Sn}{}^{⁗}+Ni^{••}\overset{}{\underset{}{↔}}Ni_{Sn}{}^{\prime \prime }$$

where *Ni*^••^ denotes a bivalence nickel ion and *Ni_Sn_*″ indicates a Ni ion occupying the Sn site with two electrons. A presumption was made that there are *m* Sn vacancies on the surface of a single SnO_2_ QD, which adsorbs *t* ions of Ni^2+^. Thus, the interaction between the SnO_2_ QD and Ni^2+^ ion is described by combining Equations (4) and (5), as shown in Equation (6):

(6) $$mV_{Sn}+tNi^{••}+4me^{\prime }\overset{k_{1}}{\underset{k_{-1}}{↔}}(m-t)V_{Sn}{}^{⁗}+tNi_{Sn}{}^{\prime \prime }$$

where *k*_1_ and *k*_−1_ are the rate constants of the reversible reaction. Hence, Equation (7) is obtained at the equivalence state:

(7) $$k_{1}{\left[V_{Sn}\right]}^{m}{\left[Ni^{••}\right]}^{t}{\left[e^{\prime }\right]}^{4m}=k_{-1}{\left[V_{Sn}{}^{⁗}\right]}^{m-t}{\left[Ni_{Sn}{}^{\prime \prime }\right]}^{t}$$

where [X] denotes the density of X for each reactant. Therefore, the density of electrons [*e*′] can be formulated as in Equation (8):

(8) $$[e^{\prime }]={\left(\frac{k_{-1}{\left[V_{Sn}{}^{⁗}\right]}^{m-t}{\left[Ni_{Sn}{}^{\prime \prime }\right]}^{t}}{k_{1}{\left[V_{Sn}\right]}^{m}}\right)}^{1/4m}{\left[Ni^{••}\right]}^{-t/4m}$$

Considering the rate constants of *k*_1_ and *k*_−1_ follow the Arrhenius equation, they are correlated to *E_ads_*, the adsorption energy of Ni^2+^ on SnO_2_ QDs, as shown in Equations (9) and (10):

(9) $$k_{1}=A\exp(\frac{E_{ads}}{kT})$$

(10) $$k_{-1}=A\exp(-\frac{E_{ads}}{kT})$$

where *A* is pre-exponential constant and *k* and *T* are the Boltzmann constant and temperature. Thus, Equation (8) can be rewritten as in Equation (11):

(11) $$\left[e^{\prime }]={\left(\frac{{\left[V_{Sn}{}^{⁗}\right]}^{m-t}{\left[Ni_{Sn}{}^{\prime \prime }\right]}^{t}}{{\left[V_{Sn}\right]}^{m}}\right)}^{1/4m}{\left[Ni^{••}\right]}^{-t/4m}\exp(-\frac{E_{ads}}{2mkT}\right)$$

It is noted that the fluorescence emission results from the recombination of electrons and holes, as expressed in Equation (12):

(12) $$h^{•}+e^{\prime }\overset{k_{2}}{\underset{k_{-2}}{↔}}h\nu$$

where *k*_2_ and *k*_−2_ are the rate constants and *hν* represents an emitted photon. Therefore, the fluorescence emission intensity (*F*) is proportional to the reaction rate of Equation (12). If *α* is the coefficient of the proportional correlation, Equation (13) can be obtained:

(13) $$F=\alpha k_{2}[h^{•}][e^{\prime }]$$

Therefore, the expression of *F* is obtained as Equation (14) and its logarithmic form is shown in Equation (15):

(14) $$F=\alpha k_{2}[h^{•}]{\left(\frac{{\left[V_{Sn}{}^{⁗}\right]}^{m-t}{\left[Ni_{Sn}{}^{\prime \prime }\right]}^{t}}{{\left[V_{Sn}\right]}^{m}}\right)}^{1/4m}{\left[Ni^{••}\right]}^{-t/4m}\exp(-\frac{E_{ads}}{2mkT})$$

(15) $$\ln F=-\frac{t}{4m}\ln[Ni^{••}]+\ln\alpha k_{2}[h^{•}]+\frac{1}{4m}\ln{\left[V_{Sn}{}^{⁗}\right]}^{m-t}{\left[Ni_{Sn}{}^{\prime \prime }\right]}^{t}{\left[V_{Sn}\right]}^{-m}-\frac{E_{ads}}{2mkT}$$

It is obvious that ln*F* is of linear dependence with logarithmic Ni^2+^ concentration provided that other parameters are constants. The slope of the linear correlation (*n*) could be found according to Equation (16). It infers that the slope is determined by the number of Sn vacancies and adsorbed Ni^2+^ ions on the SnO_2_ QD surface.

(16) $$n=\frac{d\ln F}{d\ln[Ni^{••}]}=-\frac{t}{4m}$$

**Figure 9:**
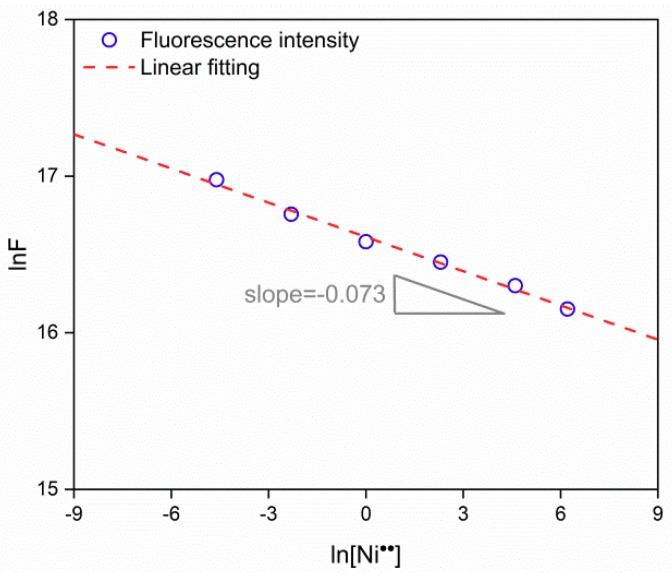
Logarithmic correlation of fluorescence emission intensity against Ni^2+^ concentration.

Figure 9 shows the correlation of fluorescence emission intensity against Ni^2+^ concentration in the logarithmic coordinates. The slope (*n*) is evaluated to be −0.073 from the linear fitting. Thus, *t*/*m* is equal to 0.29. It means that 71% of Sn vacancies on the surface are left vacant, while 29% of them are active to Ni^2+^ ions. Therefore, the fluorescence response of SnO_2_ QDs to heavy metal ions is controlled by the density of active Sn vacancies. Furthermore, it is likely to be correlated to several factors, such as adsorption energy and concentration of heavy metal ions as well as total density of surface Sn defects. It is known that *n* is proportional to *t*/*m*, where *t* and *m* are the number of Ni^2+^ ions and Sn vacancies on the surface. If Ni^2+^ is replaced by another metal ion, Equation (16) is still valid. In this case, however, *F* will be influenced, because its *E_ads_* value changes in Equation (15). Thus, the present mechanism of fluorescence response could be general for the detection of a series of heavy metal ions.

## 4. Conclusions

The SnO_2_ QDs were prepared in the aqueous solution by the hydrolysis and oxidation of SnCl_2_ source material. The average grain size of the QDs was 2.23 nm from HRTEM observation. The rutile phase SnO_2_ of the prepared QDs was confirmed from the XRD pattern, HRTEM observation and XPS spectrum. The fluorescence spectra of the SnO_2_ QDs show intensity peaked at an emission wavelength of 300 nm. The SnO_2_ QDs showed a fluorescence response to various heavy metal ions and the response of 2.48 was observed to be 100 ppm Ni^2+^. The fluorescence performance to Ni^2+^ were evaluated in the background solutions of deionized water, deionized water with Fe^3+^ ions, reclaimed water and sea water. The limit of detection was as low as 0.01 ppm for Ni^2+^. The prepared QDs showed a great potential in the sensor development for the detection of heavy metal ions in contaminated water. The first principle calculation demonstrated that Ni^2+^ and Fe^3+^ had an adsorption energy of 7.65 and 13.46 eV, respectively. The fluorescence response was found to be dependent on the adsorption energy as well as ion radius of heavy metal ions. The mechanism of fluorescence response was discussed based on the interaction between Sn vacancies and Ni^2+^ ions. The fluorescence emission intensity was formulated as a function of Ni^2+^ concentration. 71% of Sn vacancies on the surface were left vacant, while 29% of them are active to Ni^2+^ ions. The density of active Sn vacancies was the crucial factor that determined the fluorescence response of SnO_2_ QDs to heavy metal ions.

## Figures and Tables

**Table 1:** Adsorption energies of heavy metal ions from first principle calculation based on the density function theory.

Heavy Metal Ion	Adsorption Energy (eV)	Ion Radius (Å)	Fluorescence Response
Cd^2+^	4.21	78	1.14
Fe^3+^	13.46	64.5	2.13
Ni^2+^	7.65	69	2.48
Pb^2+^	5.39	119	1.03
